# Hematopoietic stem cell transplantation for DLBCL: a report from the European Society for Blood and Marrow Transplantation on more than 40,000 patients over 32 years

**DOI:** 10.1038/s41408-024-01085-9

**Published:** 2024-07-05

**Authors:** Philipp Berning, Mathilde Fekom, Maud Ngoya, Anthony H. Goldstone, Peter Dreger, Silvia Montoto, Hervé Finel, Evgenii Shumilov, Patrice Chevallier, Didier Blaise, Tim Strüssmann, Ben Carpenter, Edouard Forcade, Cristina Castilla-Llorente, Marek Trneny, Hervé Ghesquieres, Saveria Capria, Catherine Thieblemont, Igor Wolfgang Blau, Ellen Meijer, Annoek E. C. Broers, Anne Huynh, Denis Caillot, Wolf Rösler, Stephanie Nguyen Quoc, Jörg Bittenbring, Arnon Nagler, Jacques-Emmanuel Galimard, Bertram Glass, Anna Sureda, Norbert Schmitz

**Affiliations:** 1 Department of Hematology and Oncology, University Hospital Muenster, Muenster, Germany; 2 European Society for Blood and Marrow Transplantation, Paris, France; 3 HCA Healthcare, Macmillan Cancer Centre, London, United Kingdom; 4 Department of Medicine V, University of Heidelberg, Heidelberg, Germany; 5 St. Bartholomew’s Hospital, Barts Health NHS Trust, London, United Kingdom; 6 Department of Hematology, CHU Nantes, Nantes, France; 7 Transplantation and Cellular Immunotherapy Program, Department of Hematology, Instititut Paoli Calmettes, MSC Lab, Aix Marseille University, Marseille, France; 8 Department of Hematology, Oncology and Stem Cell Transplantation, University of Freiburg Medical Center, Freiburg, Germany; 9 Department of Hematology, University College London Hospitals, London, United Kingdom; 10 Service d’Hématologie Clinique et Thérapie Cellulaire, CHU Bordeaux, F-33000 Bordeaux, France; 11 Department of Hematology, Gustave Roussy Cancer Campus, Villejuif, France; 12 First Faculty of Medicine, Charles University, Praha, Czech Republic; 13 Hospices Civils de Lyon, Hôpital Lyon Sud, Service d’Hématologie, Pierre Bénite, France; 14 Department of Translational and Precision Medicine, Policlinico Umberto I Hospital, Sapienza University of Rome, Rome, Italy; 15 Department of Hemato-Oncology, Hôpital Saint-Louis APHP, Paris, France; 16 Charité-Universitätsmedizin Berlin, Freie Universität Berlin and Humboldt-Universität Berlin, Department of Hematology, Oncology and Tumor Immunology, Campus Virchow Clinic, Berlin, Germany; 17 Department of Hematology, Amsterdam University Medical Center, Free University, Amsterdam, the Netherlands; 18 Department of Hematology, Erasmus MC Cancer Institute, Rotterdam, Netherlands; 19 CHU - Institut Universitaire du Cancer Toulouse, Oncopole, I.U.C.T-O, Toulouse, France; 20 Department of Hematology, CHU Dijon, Dijon, France; 21 Department of Internal Medicine 5, Hematology and Oncology, University of Erlangen-Nuremberg, Erlangen, Germany; 22 Department of Hematology, AP-HP, Sorbonne Université, Pitié- Salpêtrière Hospital, Paris, France; 23 Department of Hematology and Oncology, Saarland University Medical School, Homburg, Germany; 24 Division of Hematology, Sheba Medical Center, Tel Hashomer, Israel; 25 Department of Hematology, Oncology, Tumor Immunology, and Palliative Care, Helios Klinikum Berlin-Buch, Berlin, Germany; 26 Department of Hematology, Institut Català d’Oncologia Hospitalet, IDIBELL, Universitat de Barcelona, Barcelona, Spain

**Keywords:** B-cell lymphoma, Disease-free survival

## Abstract

Autologous(auto-) and allogeneic(allo-) hematopoietic stem cell transplantation (HSCT) are key treatments for relapsed/refractory diffuse large B-cell lymphoma (DLBCL), although their roles are challenged by CAR-T-cells and other immunotherapies. We examined the transplantation trends and outcomes for DLBCL patients undergoing auto-/allo-HSCT between 1990 and 2021 reported to EBMT. Over this period, 41,148 patients underwent auto-HSCT, peaking at 1911 cases in 2016, while allo-HSCT saw a maximum of 294 cases in 2018. The recent decline in transplants corresponds to increased CAR-T treatments (1117 cases in 2021). Median age for auto-HSCT rose from 42 (1990–1994) to 58 years (2015–2021), with peripheral blood becoming the primary stem cell source post-1994. Allo-HSCT median age increased from 36 (1990–1994) to 54 (2015–2021) years, with mobilized blood as the primary source post-1998 and reduced intensity conditioning post-2000. Unrelated and mismatched allo-HSCT accounted for 50% and 19% of allo-HSCT in 2015–2021. Three-year overall survival (OS) after auto-HSCT improved from 56% (1990–1994) to 70% (2015–2021), *p* < 0.001, with a decrease in relapse incidence (RI) from 49% to 38%, while non-relapse mortality (NRM) remained unchanged (4%). After allo-HSCT, 3-year-OS increased from 33% (1990–1999) to 46% (2015–2021) (*p* < 0.001); 3-year RI remained at 39% and 1-year-NRM decreased to 19% (*p* < 0.001). Our data reflect advancements over 32 years and >40,000 transplants, providing insights for evaluating emerging DLBCL therapies.

## Introduction

The first reports on autologous and allogeneic transplantation of hematopoietic stem cells performed in end-stage patients with relapsed or refractory aggressive lymphoma were published in the early 1990s [1–6]. The basic principles of transplantation first described in patients with leukemia were shown to also apply to patients with lymphoma: the superior anti-tumor effect of dose-escalated chemotherapy to prepare patients for transplantation, better outcomes of patients with chemo-sensitive as compared to chemo-refractory disease, and the favorable survival of patients transplanted in complete or partial remission as opposed to patients being refractory to salvage chemotherapy [7–9]. Results of allogeneic bone marrow transplantation suggested the existence of a graft-versus-lymphoma effect (GvL) comparable to the graft-versus-leukemia effect described earlier [10]. The albeit limited success of allo-HSCT in patients with completely chemorefractory disease and the ability of donor lymphocyte infusions (DLI) to induce further remissions in patients relapsing after allo-HSCT have repeatedly been taken as an evidence of GvL [11–15]. Further relying on the therapeutic potential of donor T-cells, conditioning shifted from myeloablative conditioning (MAC) to reduced-intensity conditioning (RIC) especially in patients with chemosensitive disease and low tumor burden [12, 13, 16–19]. RIC also resulted in expanded access to allo-HSCT for older and frail patients [12, 13, 17]. The switch from bone marrow to G-CSF-mobilized blood as the preferred stem cell source, first reported for auto-HSCT [20, 21] and later for allo-HSCT [22], also contributed to the steep increase of transplant numbers observed for all lymphomas in the new millennium [23]. Large donor registries and the adoption of haplo-identical transplantation helped finding a suitable donor for almost every patient within a short period of time. Unfortunately, despite all progress made in donor-recipient matching, conditioning, GvHD prophylaxis, and supportive care, allo-HSCT remains loaded with a relatively high treatment-related mortality (TRM), primarily due to graft-versus-host disease [13, 16].

In recent years, transplantation of B-cell lymphoma patients has come under scrutiny by the development of CD19-directed chimeric antigen receptor (CAR) T-cells which show promising efficacy without the high TRM typical for allo-HSCT [24–27].

Here, we describe the pivotal changes in transplant modalities and outcomes of auto- and allo-HSCT for diffuse large B-cell lymphoma (DLBCL) in a very large cohort of patients registered with the European Society for Blood and Marrow Transplantation (EBMT) over 32 years. These benchmarking data will help to assess new therapeutic strategies for patients with DLBCL.

## Methods

### Data collection

We conducted a retrospective analysis of patients registered with the EBMT. Details describing the data collection process, quality management, and data hosting have been published previously [28]. All participating institutions are required to obtain written informed consent from patients prior to registration with EBMT, following the current version of the Helsinki Declaration. Numbers of auto-HSCT, allo-HSCT, and CAR T-cell infusions performed in adult patients with DLBCL between 1990 and 2021 were collected from centers reporting to the EBMT. For CAR T-cells, numbers were available from 2016 to 2021. Adult patients ≥18 years diagnosed with DLBCL receiving auto-HSCT as a first transplant or allo-HSCT as first transplant or after previous auto-HSCT, grafted between 1990 and 2021 were identified in the EBMT registry. For this analysis, patients with DLBCL were considered, including germinal center B-cell type (GCB) DLBCL, activated B-cell type (ABC or non-GCB) DLBCL, DLBCL not otherwise specified (NOS), primary cutaneous DLBCL, EBV-positive DLBCL, DLBCL associated with chronic inflammation, intravascular large B-cell lymphoma, ALK-positive large B-cell lymphoma, Human Herpesvirus-8 (HHV8) positive DLBCL, NOS as well as additional DLBCL-related subtypes as shown in Fig. S1. Eligible patients were registered by 578 transplant centers. Over the 32-year period from 1990 to 2021, the process of data reporting to EBMT evolved in accordance with national and center-specific regulations. EBMT members are generally required to report all cases, but consistent data reporting is the responsibility of individual centers. Consequently, we are unable to report to which extent the number of cases reported to EBMT reflects the number of cases actually transplanted in the countries covered by our report and how the number of reported compared to actually transplanted cases may have changed over time. Centers contributing 200 or more patients are listed in Table S1.

### Definitions

Diagnosis of DLBCL was based on local pathology review using criteria effective at the time of diagnosis. Disease stage was classified according to the Ann Arbor staging system. Refractory disease was defined as disease progressing during first-line (immuno-)chemotherapy or in patients with transient response [complete (CR) or partial response (PR) lasting ≤3 months] after induction treatment. Relapse was diagnosed in case of lymphoma recurrence occurring at least 3 months after end of therapy in patients having achieved CR. Disease status was assessed by individual investigators according to standard criteria at the time patients were referred for transplantation and classified as CR, PR, stable disease (SD) and progressive disease (PD). Regimens containing TBI > 6 Gy, total oral busulfan >8 mg/kg or a total of intravenous busulfan >6.4 mg/kg body weight were classified as myeloablative, all other regimens were classified as reduced intensity conditioning (RIC) [29]. The diagnosis and grading of acute GvHD (aGvHD) and chronic GvHD (cGvHD) were made by transplant centers according to established criteria [30, 31].

### Statistical analysis

Endpoints analyzed were progression-free survival (PFS) defined as survival without lymphoma relapse or progression, overall survival (OS) defined as time from transplantation to death from any cause; non-relapse mortality (NRM) defined as death without previous lymphoma relapse and relapse incidence (RI) defined as disease recurrence after transplantation. In patients receiving allo-HSCT, the incidence and severity of aGvHD and cGvHD were analyzed. All outcomes were measured from the day of transplantation. Surviving patients were censored at the time of last contact. The probabilities of OS and PFS were calculated using the Kaplan–Meier method. We calculated cumulative incidences for RI and NRM using a competing risk model, where death was treated as a competing event for relapse. Death and relapse were considered as competing events for aGvHD and cGvHD. Demographics were compared between groups using the chi- squared test or Fisher’s exact test for categorical variables and the Mann–Whitney U test for continuous variables. Univariate analyses were performed using the log-rank test for PFS and OS, while Gray’s test was used for competing risk outcome data. Multivariate analyses were performed using the Cox proportional-hazards regression model. Results were reported as hazard ratios (HR) with a 95% confidence interval (95% CI). All statistical tests were two-sided with a type I error fixed at 0.05 for factors associated with time-to-event outcomes. All analyses were performed using R version 4.3.3 with the R packages survival version 3.5-8, cmprsk version 2.2-11 and Hmisc version 5.1-2. (R Core Team. R: a language for statistical computing. 2014. R Foundation for Statistical Computing, Vienna, Austria).

## Results

### Evolution of transplantation and CAR T-cell numbers over time

**Fig. 1:**
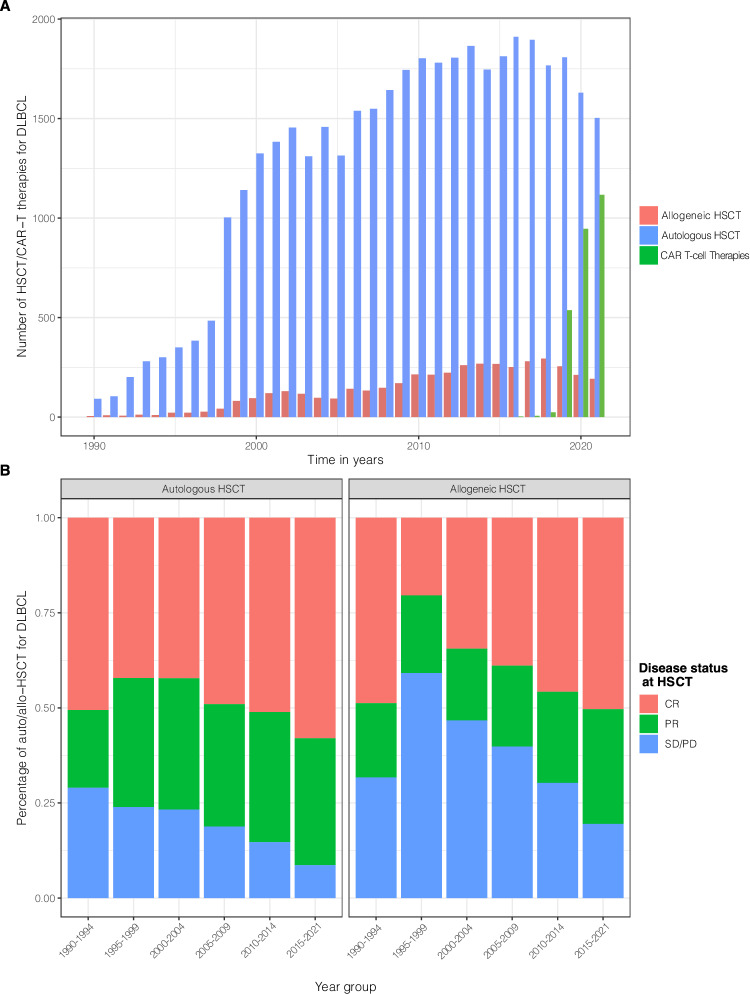
Trends for hematopoietic stem cell transplantation and CAR T-cell infusions for DLBCL over time. Numbers of auto- and allo-HSCT (**A**) by indicated periods between 1990 and 2021. The proportion (**B**) of patients undergoing auto-HSCT and allo-HSCT in CR/PR or SD/PD. Auto-/allo-HSCT autologous/allogeneic hematopoietic stem cell transplantation, CR complete remission, PR partial remission, SD stable disease, PD progressive disease.

In total, 41,148 auto-HSCTs and 4562 allo-HSCTs for DLBCL meeting the inclusion criteria were registered with EBMT between 1990 and 2021. As shown in Fig. 1A, transplantation activities constantly increased over time. Annual numbers of auto-HSCT increased from 92 in 1990 to reach a maximum of 1911 in 2016; allo-HSCT increased from 5 in 1990 to 294 in 2018. After 2018, the numbers of auto-HSCT and allo-HSCT sharply declined with no more than 1503 and 192 HSCT reported for 2021, respectively. Conversely, the number of CAR T-cell infusions steeply increased since 2016 (3 CAR T-cell therapies reported to EBMT) to 1117 in 2021 (Fig. 1A).

Detailed clinical data were available for 43,260 DLBCL patients, of which 41,148 received an auto-HSCT and 4562 underwent allo-HSCT (2450 patients received allo-HSCT after auto-HSCT). The number of reporting centers as well as the number of patients per center increased over time. The number of patients receiving auto-HSCT in CR or PR increased from 681 (71%) between 1990-1994 to 11,287 patients in the most recent period accounting for 91% of all auto-HSCT (Fig. 1B). Similar trends were observed for patients undergoing allo-HSCT.

### Auto-HSCT: patient characteristics and outcomes

The age and the proportion of patients with good performance status undergoing auto-HSCT significantly increased from 42 years (range: 18–67) to 58 years (range: 49–65) (*p* < 0.001) and from 90% to 94% (*p* < 0.001) for the time periods 1990–1994 and 2015–2021, respectively (Table 1). After 1994, peripheral blood (PB) emerged as the universally used stem cell source, accounting for more than 97% of all auto-HSCT between 1990 and 2021. TBI for conditioning has practically been abandoned after 2005 (Table 1). Preparation with BEAM or similar regimens remained most popular throughout all time periods (Table 1). Rituximab as part of conditioning was first reported after 2000 with 7.2% of all patients receiving Rituximab between 2015 and 2021 (Table 1). Significantly higher proportions of patients in CR or PR than in SD or PD were autografted over time (*p* < 0.001) (Fig. 1B, Table 1).

**Table 1:** Baseline characteristics for auto-HSCT patients over time.

Characteristics	Overall	Time Periods						
		1990–1994	1995–1999	2000–2004	2005–2009	2010–2015	2015–2021	*P* value
**No. HSCT**	41,148	977	3364	6932	7794	9037	13,044	
**No. of centers**	578	147	345	416	432	448	471	
**Median age at HSCT in years (IQR) [range]**	54 (44, 62) [18, 85]	42 (33, 50) [18, 67]	48 (37, 55) [18, 78]	51 (40, 58) [18, 85]	54 (44, 61) [18, 84]	56 (46, 63) [18, 80]	58 (49, 65) [18, 82]	<0.001
**Patient Gender**								0.012
Male	24,627 (60%)	567 (58%)	1956 (58%)	4064 (59%)	4675 (60%)	5465 (61%)	7900 (61%)	
Female	16,451 (40%)	410 (42%)	1405 (42%)	2859 (41%)	3098 (40%)	3554 (39%)	5125 (39%)	
Missing	70	0	3	9	21	18	19	
**Median time diagnosis – HSCT in months (IQR) [range]**	12 (6, 23) [0, 485]	10 (5, 19) [2, 268]	10 (6, 20) [0, 376]	10 (6, 19) [1, 370]	12 (7, 24) [0, 485]	12 (7, 25) [0. 374]	12 (7, 24) [0, 407]	<0.001
**Time diagnosis to HSCT**								<0.001
0-12 months	21,084 (52%)	559 (59%)	1911 (57%)	3976 (58%)	3951 (51%)	4407 (49%)	6280 (48%)	
>12 months	19,805 (48%)	392 (41%)	1420 (43%)	2857 (42%)	3793 (49%)	4611 (51%)	6732 (52%)	
Missing	259	26	33	99	50	19	32	
**Number of treatment lines before HSCT**								
≤1 line	7429 (35%)	634 (78%)	1070 (65%)	1312 (47%)	515 (29%)	593 (26%)	3305 (28%)	
2 lines	9555 (46%)	146 (18%)	458 (28%)	1059 (38%)	858 (48%)	1093 (50%)	5941 (51%)	
≥3 lines	3937 (19%)	29 (3.6%)	111 (6.8%)	388 (14%)	416 (23%)	521 (24%)	2472 (21%)	
Missing	20,227	168	1725	4173	6005	6830	1326	
**Disease status at HSCT**								<0.001
CR	19,752 (51%)	485 (51%)	1341 (42%)	2689 (42%)	3628 (49%)	4446 (51%)	7163 (58%)	
PR	12,954 (33%)	196 (20%)	1080 (34%)	2198 (35%)	2379 (32%)	2977 (34%)	4124 (33%)	
SD/PD	6254 (16%)	278 (29%)	761 (24%)	1482 (23%)	1391 (19%)	1277 (15%)	1065 (8.6%)	
Missing	2188	18	182	563	396	337	692	
**KPS at HSCT**								<0.001
< 80%	2115 (6.3%)	12 (9.9%)	135 (8.5%)	298 (5.8%)	469 (6.6%)	465 (5.7%)	736 (6.3%)	
≥ 80%	31,678 (94%)	109 (90%)	1453 (91%)	4843 (94%)	6650 (93%)	7670 (94%)	10,953 (94%)	
Missing	7355	856	1776	1791	675	902	1355	
**Rituximab as part of any treatment**								<0.001
No	5810 (31%)	351 (100%)	1021 (100%)	1787 (81%)	242 (13%)	230 (9.1%)	2179 (20%)	
Yes	13,232 (69%)	0 (0%)	1 (<0.1%)	407 (19%)	1591 (87%)	2290 (91%)	8943 (80%)	
Missing	22,106	626	2342	4738	5961	6517	1922	
**Stem cell source**								<0.001
BM	963 (2.4%)	522 (53%)	174 (5.2%)	109 (1.6%)	80 (1.0%)	46 (0.5%)	32 (0.2%)	
PB	39,599 (97%)	378 (39%)	3108 (93%)	6711 (98%)	7631 (98%)	8922 (99%)	12,849 (100%)	
BM+PB	311 (0.8%)	76 (7.8%)	68 (2.0%)	62 (0.9%)	58 (0.7%)	24 (0.3%)	23 (0.2%)	
Missing	275	1	14	50	25	45	140	
**Conditioning regimens**								
BEAM or similar	23,786 (73%)	513 (63%)	1127 (62%)	2023 (68%)	4457 (83%)	6936 (79%)	8730 (69%)	
Other	4831 (15%)	212 (26%)	570 (31%)	521 (18%)	753 (14%)	1124 (13%)	1651 (13%)	
BCNU+thiotepa	1395 (4.3%)	0 (0%)	1 (<0.1%)	4 (0.1%)	88 (1.6%)	362 (4.1%)	940 (7.4%)	
BENDA+EAM	1038 (3.2%)	0 (0%)	0 (0%)	0 (0%)	5 (<0.1%)	171 (2.0%)	862 (6.8%)	
CBV	753 (2.3%)	82 (10%)	116 (6.4%)	425 (14%)	59 (1.1%)	43 (0.5%)	28 (0.2%)	
TEAM	670 (2.1%)	1 (0.1%)	4 (0.2%)	1 (<0.1%)	25 (0.5%)	113 (1.3%)	526 (4.1%)	
Missing	8675	169	1546	3958	2407	288	307	
**TBI for conditioning**								<0.001
No	39,639 (98%)	776 (84%)	2986 (93%)	6472 (98%)	7616 (99%)	8917 (99%)	12,872 (99%)	
Yes	768 (1.9%)	153 (16%)	226 (7.0%)	157 (2.4%)	88 (1.1%)	70 (0.8%)	74 (0.6%)	
Missing	741	48	152	303	90	50	98	
**Rituximab as part of conditioning**								
No	30,529 (94%)	808 (100%)	1818 (100%)	2947 (99%)	5095 (95%)	8047 (92%)	11,814 (93%)	
Yes	1944 (6.0%)	0 (0%)	0 (0%)	27 (0.9%)	292 (5.4%)	702 (8.0%)	923 (7.2%)	
Missing	8675	169	1546	3958	2407	288	307	

*No*. number, *HSCT* hematopoietic stem cell transplantation, *IQR* interquartile range, *CR* complete remission, *PR* partial remission, *SD* stable disease, *PD* progressive disease, *KPS* Karnofsky performance score, *PB* peripheral blood, *BEAM* carmustine (BCNU), etoposide, cytarabine, melphalan, *TEAM* thiotepa, etoposide, cytarabine, melphalan, *Benda+EAM* bendamustine, etoposide, cytarabine, melphalan; *CBV* cyclophosphamide, carmustine, etoposide, *TBI* total body irradiation.

**Fig. 2:**
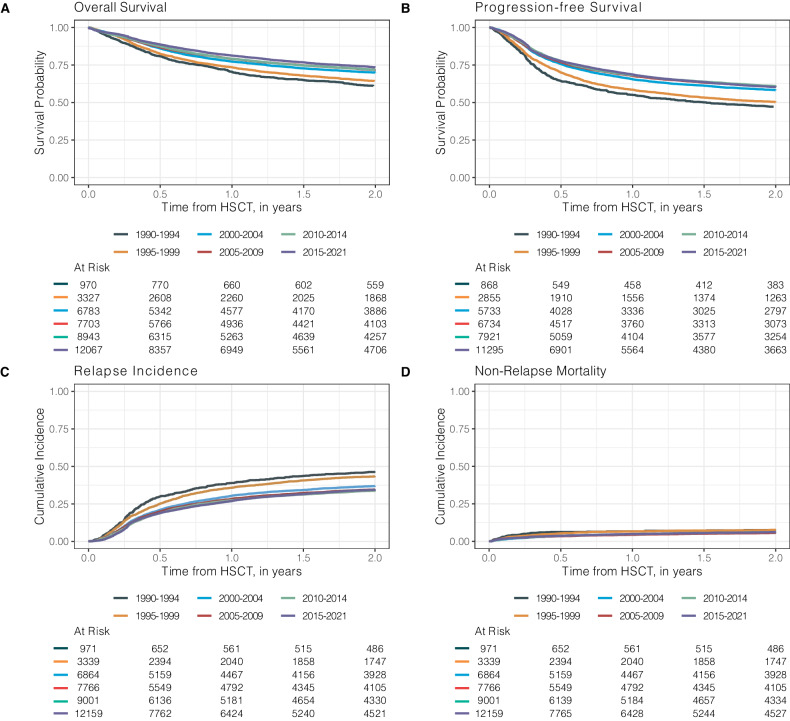
Kaplan-Meier estimates of auto-HSCT outcomes over time. Overall survival (**A**), progression-free survival (**B**), cumulative incidence of relapse (**C**) and non-relapse mortality (**D**) after auto-HSCT by indicated periods between 1990 and 2021. Auto-HSCT autologous hematopoietic stem cell transplantation.

Major outcomes of patients receiving auto-HSCT are shown in Fig. 2 and Table 2. With a median follow-up of 4.7 years (95% CI: 4.7–4.8 years), 1-year, 5-year, and 10-year overall survival (OS) rates were 78.6% (95% CI: 78.2–79.1%), 61.1% (95% CI: 60.4–61.7%), and 52.6% (95% CI: 51.8–53.4%). We noted significant improvements in OS and PFS over time reaching 3-year OS and PFS rates of 69.5% (95% CI: 68.4–70.5%) and 55.9% (95% CI: 54.8–57.0%) for the 2015-2021 period (Table 2). Three-year relapse rates significantly decreased form 49.4% (95% CI: 46–52.7) for the 1990-1994 period to 38.0% (95% CI: 36.9–39.0%) in the 2015-2021 period. One-year NRM remained at 4–6% throughout the entire study period despite slight improvements over time (Fig. 2, Table 2).

**Table 2:** Outcomes after auto-HSCT by time periods.

Outcomes after auto-HSCT in % (95% CI)									
Variable	Time	Overall	Time Periods						
			1990–1994	1995–1999	2000–2004	2005–2009	2010–2015	2015–2021	*P* value
NRM	1 year	4.4 (4.2–4.6)	6.0 (4.6–7.7)	5.7 (4.9–6.6)	4.1 (3.6–4.6)	3.9 (3.5–4.4)	4.3 (3.9–4.8)	4.4 (4–4.8)	0.002
	3 years	5.9 (5.6–6.2)	7.0 (5.4–8.8)	6.8 (5.9–7.8)	5.3 (4.8–6)	5.5 (5–6.1)	5.9 (5.4–6.5)	6.1 (5.6–6.6)	
RI	1 year	29 (28.5–29.5)	39 (35.7–42.2)	35.7 (34–37.5)	30.4 (29.2–31.7)	28.4 (27.2–29.5)	27.5 (26.4–28.5)	26.9 (26–27.8)	< 0.001
	3 years	39.1 (38.6–39.7)	49.4 (46–52.7)	46.5 (44.6–48.3)	39.8 (38.5–41.1)	37.9 (36.6–39.1)	37.2 (36–38.4)	38.0 (36.9–39.0)	
PFS	1 year	66.6 (66.1–67.1)	55 (51.6–58.3)	58.5 (56.7–60.4)	65.5 (64.2–66.7)	67.7 (66.5–68.9)	68.2 (67.1–69.3)	68.7 (67.8–69.6)	< 0.001^b^
	2 years	58.9 (58.3–59.4)	47.3 (43.9–50.6)	50.2 (48.3–52.1)	58.2 (56.8–59.5)	60.6 (59.3–61.8)	60.9 (59.7–62.1)	60.3 (59.3–61.3)	
	3 years	55 (54.4–55.6)	43.6 (40.2–46.9)	46.7 (44.8–48.6)	54.9 (53.5–56.2)	56.6 (55.3–57.9)	56.9 (55.6–58.1)	55.9 (54.8–57.0)	
	5 years	49.5 (48.8–50.2)	38.3 (35–41.6)	41.6 (39.7–43.5)	50.6 (49.2–51.9)	51.9 (50.6–53.3)	51 (49.7–52.3)	^a^	
	10 years	41.5 (40.6–42.4)	32.4 (29.1–35.8)	35.4 (33.5–37.3)	43.4 (41.9–44.8)	43.8 (42.4–45.3)	^a^	^a^	
OS	1 year	78.6 (78.2–79.1)	70.5 (67.5–73.3)	73.5 (71.9–75)	77.3 (76.2–78.3)	79.1 (78.1–80)	79.2 (78.2–80.1)	81.3 (80.5–82)	< 0.001^b^
	2 years	70.9 (70.4–71.4)	61 (57.8–64.1)	64.4 (62.7–66)	70 (68.8–71.1)	71.8 (70.6–72.8)	71.8 (70.7–72.8)	73.5 (72.5–74.4)	
	3 years	66.9 (66.4–67.4)	55.6 (52.3–58.7)	60.2 (58.4–61.9)	66.3 (65.1–67.5)	67.8 (66.7–69)	67.7 (66.6–68.8)	69.5 (68.4–70.5)	
	5 years	61.1 (60.4–61.7)	50.7 (47.4–53.9)	55.5 (53.7–57.3)	62.3 (61–63.5)	63 (61.8–64.2)	62.1 (60.9–63.3)	^a^	
	10 years	52.6 (51.8–53.4)	43.9 (40.5–47.2)	48.7 (46.8–50.6)	54.5 (53.1–55.8)	53.9 (52.5–55.3)	^a^	^a^	

*CI* confidence interval, *NRM* non-relapse mortality, *RI* relapse incidence, *PFS* Progression-free Survival, *OS* Overall Survival

^a^Outcomes were not available due to follow-up.

^b^*P* value representing outcomes with available data.

Figure S2A illustrates outcomes of patients who underwent auto-HSCT for consolidation after achieving a CR following one treatment line compared to two and more treatment lines. Two-year OS and PFS of patients transplanted after one treatment line were significantly better than in patients undergoing auto-HSCT after two or more lines of therapy [3-year OS: 79.3% (95% CI: 77.8–80.8%) vs. 72.6.7% (95% CI: 71.1–74.1%) vs. 69.1% (95% CI: 66.2–71.1%); 3-year PFS: 68.3% (95% CI: 66.5–70.1%) vs. 58.6% (95% CI: 56.9–60.3%) vs. 52.0% (95% CI: 48.9–55.0%); *p* < 0.001 for both]. This difference was primarily due to a lower relapse incidence in patients who underwent consolidative auto-HSCT (*p* < 0.001). As shown in Fig. S2B, patients who underwent auto-HSCT not being in CR had significantly inferior OS and PFS compared to CR patients after auto-HSCT (both *p* < 0.001).

To further investigate the effect of remission status and conditioning before auto-HSCT, we used a multivariate model including the following variables with known prognostic relevance: year of HSCT, age at HSCT, gender, time interval from HSCT ≤ 12 months, disease status at HSCT, and type of conditioning regimen (Table S2). For Auto-HSCT patients, not being in CR was associated with lower OS and PFS rates, as well as being treated before 2000 compared to after 2000, similarly a longer interval from diagnosis to HSCT ( > 12 months) was associated with inferior OS and PFS compared to patients transplanted within 12 months from diagnosis (all *p* < 0.001) (Table S2). These differences in OS and PFS were primarily influenced by an increased relapse risk in patients transplanted not in CR (*p* < 0.001), patients who underwent auto-HSCT before 2000 [1995–1999 period: HR = 1.25 (95% CI: 1.14–1.37) compared to 2015–2021 period as reference, *p* < 0.001], as well as receiving conditioning therapy other than BEAM [HR = 1.11 (95% CI: 1.06–1.17), *p* < 0.001], or a longer treatment interval from diagnosis ( > 12 months) [HR = 1.20 (95% CI: 1.15–1.26), *p* < 0.001].

### Allo-HSCT: patient characteristics and outcomes

Major clinical characteristics of allografted patients are shown in Table 3. Patient age significantly increased from 36 years (range: 19–50) in 1990–1994 to 54 years (range: 18–76) in 2015–2021 (*p* < 0.001). The median time from diagnosis to allo-HSCT increased from 15 months (range 3–74) for the 1990–1994 period to 19 months (range 1–354) (*p* < 0.001) over time with an increasing proportion of patients having failed a previous auto-HSCT (47% of all allo-HSCT for 2015–2021) (Table 3). Peripheral blood became the universal source of allogeneic stem cells after 1998. The proportion of patients undergoing allo-HSCT in CR/PR significantly increased from 69% in 1990–1994 to 80% in 2015–2021 (*p* < 0.001) (Table 3). RIC was introduced in the 1990s and preceded allo-HSCT in 54% of cases in the most recent time period. In the early days, 74% of patients received TBI as part of conditioning declining to 23% in the most recent period (Table 3). Transplantations from unrelated or haploidentical/mismatched related donors increased over time; 50% and 19% of HSCT were from such donors between 2015 and 2021. The frequencies for T-cell depletion and other GVHD prophylaxis are listed in Table 3.

**Table 3:** Baseline characteristics for allo-HSCT patients over time.

Characteristics	Overall	Time Periods						
		1990–1994	1995–1999	2000–2004	2005–2009	2010–2015	2015–2021	*P* value
**No. HSCT**	4562	43	195	559	685	1186	1894	
**No. of centers**	327	28	82	172	201	245	274	
**Median age at HSCT in years (IQR) [range]**	50 (41, 58) [18, 76]	36 (28, 41) [19, 50]	39 (31, 46) [20, 75]	44 (35, 52) [18, 67]	49 (41, 56) [18, 71]	52 (43, 59) [19, 75]	54 (43, 61) [18, 76]	<0.001
**Patient Gender**								0.5
Male	2894 (63%)	29 (67%)	119 (61%)	338 (60%)	428 (62%)	760 (64%)	1220 (64%)	
Female	1666 (37%)	14 (33%)	76 (39%)	221 (40%)	257 (38%)	426 (36%)	672 (36%)	
Missing	2	0	0	0	0	0	2	
**Median time diagnosis – HSCT in months (IQR) (range)**	20 (12, 38) [1, 354]	15 (10, 21) [3, 74]	14 (9, 27) [1, 196]	21 (12, 41) [2, 218]	22 (13, 41) [2. 298]	22 (12, 43) [2, 279]	19 (11, 34) [1, 354]	<0.001
**Time diagnosis to HSCT**								<0.001
0–12 months	1165 (26%)	13 (32%)	76 (40%)	131 (24%)	136 (20%)	281 (24%)	528 (28%)	
>12 months	3379 (74%)	28 (68%)	116 (60%)	425 (76%)	543 (80%)	904 (76%)	1363 (72%)	
Missing	18	2	3	3	6	1	3	
**Previous auto-HSCT**								<0.001
No	2112 (46%)	36 (84%)	138 (71%)	249 (45%)	261 (38%)	432 (36%)	996 (53%)	
Yes	2450 (54%)	7 (16%)	57 (29%)	310 (55%)	424 (62%)	754 (64%)	898 (47%)	
**Disease status at HSCT**								
CR	1899 (44%)	20 (49%)	38 (20%)	177 (34%)	247 (39%)	516 (46%)	901 (50%)	<0.001
PR	1088 (25%)	8 (20%)	38 (20%)	97 (19%)	135 (21%)	270 (24%)	540 (30%)	
SD/PD	1306 (30%)	13 (32%)	110 (59%)	240 (47%)	253 (40%)	341 (30%)	349 (19%)	
Missing	269	2	9	45	50	59	104	
**KPS at HSCT**								<0.001
< 80%	362 (9.0%)	1 (9.1%)	18 (18%)	54 (12%)	83 (13%)	68 (6.1%)	138 (7.9%)	
≥ 80%	3669 (91%)	10 (91%)	81 (82%)	399 (88%)	537 (87%)	1042 (94%)	1600 (92%)	
Missing	531	32	96	106	65	76	156	
**Rituximab as part of any treatment**								<0.001
No	678 (27%)	18 (100%)	72 (100%)	172 (79%)	39 (18%)	47 (12%)	330 (21%)	
Yes	1797 (73%)	0 (0%)	0 (0%)	47 (21%)	175 (82%)	347 (88%)	1228 (79%)	
Missing	2087	25	123	340	471	792	336	
**Stem cell source**								<0.001
BM	534 (12%)	41 (95%)	81 (42%)	102 (18%)	61 (9.2%)	100 (8.7%)	149 (7.9%)	
PB	3930 (88%)	1 (2.3%)	111 (57%)	447 (81%)	598 (90%)	1046 (91%)	1727 (92%)	
BM+PB	18 (0.4%)	1 (2.3%)	2 (1.0%)	4 (0.7%)	4 (0.6%)	2 (0.2%)	5 (0.3%)	
Missing	80	0	1	6	22	38	13	
**Conditioning intensity**								<0.001
RIC	2401 (55%)	0 (0%)	36 (26%)	289 (56%)	397 (59%)	671 (58%)	1008 (54%)	
MAC	1981 (45%)	33 (100%)	101 (74%)	228 (44%)	275 (41%)	489 (42%)	855 (46%)	
Missing	180	10	58	42	13	26	31	
**TBI for conditioning**								
>6 Gy	495 (11%)	30 (75%)	61 (38%)	53 (12%)	71 (11%)	111 (9.5%)	169 (9.0%)	
≤ 6 Gy	519 (12%)	0 (0%)	3 (1.9%)	27 (5.9%)	72 (11%)	151 (13%)	266 (14%)	
No TBI	3334 (77%)	10 (25%)	98 (60%)	374 (82%)	499 (78%)	910 (78%)	1443 (77%)	
Missing	214	3	33	105	43	14	16	
**Conditioning chemotherapy**								
Bu/Flu based	1213 (29%)	0 (0%)	8 (5.3%)	52 (13%)	116 (20%)	362 (31%)	675 (36%)	
TBI based	1186 (28%)	33 (85%)	89 (59%)	178 (45%)	178 (31%)	268 (23%)	440 (24%)	
Flu/Mel based	717 (17%)	0 (0%)	12 (7.9%)	63 (16%)	112 (19%)	228 (20%)	302 (16%)	
Other	706 (17%)	6 (15%)	39 (26%)	69 (17%)	129 (22%)	185 (16%)	278 (15%)	
Treo based	204 (4.9%)	0 (0%)	0 (0%)	6 (1.5%)	27 (4.7%)	71 (6.2%)	100 (5.4%)	
BEAM or similar	158 (3.8%)	0 (0%)	3 (2.0%)	32 (8.0%)	17 (2.9%)	40 (3.5%)	66 (3.5%)	
Missing	378	4	44	159	106	32	33	
**Rituximab as part of conditioning**								<0.001
No	4048 (97%)	39 (100%)	151 (100%)	399 (100%)	546 (94%)	1095 (95%)	1818 (98%)	
Yes	136 (3.3%)	0 (0%)	0 (0%)	1 (0.3%)	33 (5.7%)	59 (5.1%)	43 (2.3%)	
Missing	378	4	44	159	106	32	33	
**Donor type**								<0.001
Sibling	1945 (44%)	41 (98%)	162 (85%)	390 (71%)	346 (53%)	446 (39%)	560 (30%)	
Unrelated	2002 (45%)	0 (0%)	18 (9.4%)	136 (25%)	279 (43%)	622 (54%)	947 (50%)	
Haplo-identical / Mismatched related	462 (10%)	0 (0%)	7 (3.7%)	18 (3.3%)	22 (3.4%)	65 (5.7%)	350 (19%)	
Matched other relative (non-sibling)	58 (1.3%)	1 (2.4%)	4 (2.1%)	8 (1.4%)	9 (1.4%)	12 (1.0%)	24 (1.3%)	
Missing	95	1	4	7	29	41	13	
**In vivo T-cell depletion**								<0.001
ATG	1511 (38%)	0 (0%)	18 (17%)	62 (22%)	171 (33%)	489 (43%)	771 (42%)	
Alemtuzumab	465 (12%)	0 (0%)	7 (6.5%)	52 (18%)	73 (14%)	154 (13%)	179 (9.7%)	
None	1962 (50%)	28 (100%)	83 (77%)	173 (60%)	270 (53%)	506 (44%)	902 (49%)	
Missing	624	15	87	272	171	37	42	
**PTCY**								<0.001
No	4039 (89%)	43 (100%)	195 (100%)	559 (100%)	685 (100%)	1112 (94%)	1445 (76%)	
Yes (%)	523 (11%)	0 (0%)	0 (0%)	0 (0%)	0 (0%)	74 (6.2%)	449 (24%)	
**GvHD prophylaxis**								<0.001
CsA alone	732 (19%)	6 (21%)	31 (29%)	104 (36%)	114 (22%)	213 (18%)	264 (14%)	
CsA + MTX	1179 (30%)	22 (79%)	68 (63%)	113 (39%)	176 (34%)	351 (30%)	449 (24%)	
CsA + MMF	1146 (29%)	0 (0%)	1 (0.9%)	33 (11%)	137 (27%)	343 (30%)	632 (34%)	
TAC + MMF	442 (11%)	0 (0%)	0 (0%)	0 (0%)	42 (8.2%)	119 (10%)	281 (15%)	
Other	445 (11%)	0 (0%)	8 (7.4%)	37 (13%)	45 (8.8%)	127 (11%)	228 (12%)	
Missing	618	15	87	272	171	33	40	

*No*. number, *HSCT* hematopoietic stem cell transplantation, *IQR* interquartile range, *CR* complete remission, *PR* partial remission, *SD* stable disease, *PD* progressive disease, *KPS* Karnofsky performance score, *PB* peripheral blood, *TBI* total body irradiation, *Bu* busulfan, *Flu* fludarabine, *Mel* melphalan, *Treo* treosulfan, *BEAM* carmustine(BCNU), etoposide, cytarabine, melphalan, *ATG* anti-thymocyte globulin, *PTCY* post-transplant cyclophosphamide, *GvHD* graft-versus-host disease, *CsA* cyclosporine A, *MTX* methotrexate, *MMF* mycophenolate mofetil, *TAC* tacrolimus.

**Fig. 3:**
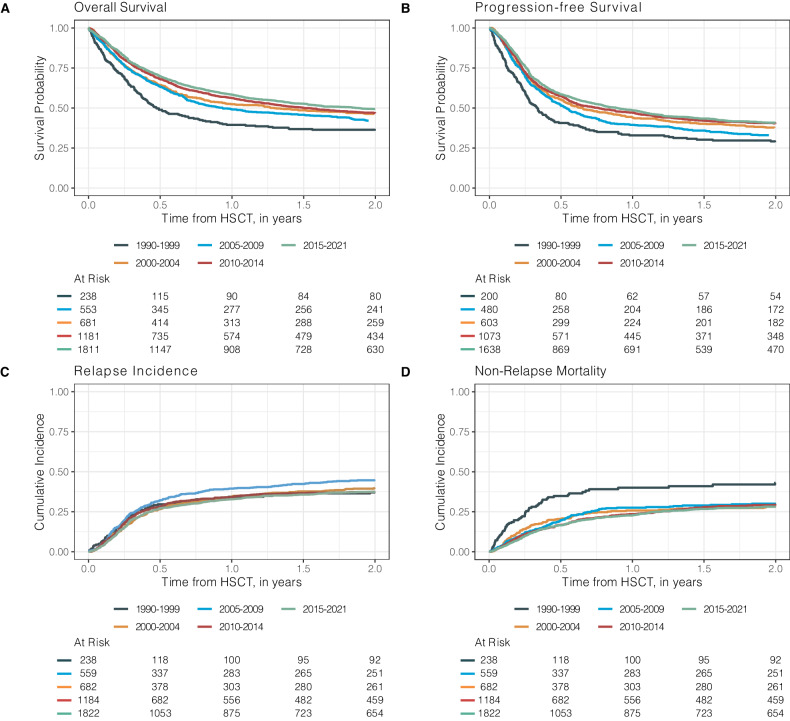
Kaplan-Meier estimates of allo-HSCT outcomes over time. Overall survival (**A**), progression-free survival (**B**), cumulative incidence of relapse (**C**) and non-relapse mortality (**D**) after allo-HSCT by indicated periods between 1990 and 2021 (outcomes for the 1990–1999 period are summarized due to low event numbers between 1990 and 1994). *Allo-HSCT* allogeneic hematopoietic stem cell transplantation.

Figure 3 and Table 4 demonstrate the improvement of key outcome parameters. With a median follow-up of 5.2 years (95% CI: 5.0–5.5 years), 3-year OS- and PFS-rates of 46.1% (95% CI: 43.6–48.6%) and 38.5% (95% CI: 36.0–41.0%) were noted for the 2015–2021 period. For the entire study period, 1-year, 5-year, and 10-year OS rates were 54.5% (95% CI: 53.0–56.0), 37.8% (95% CI: 35.8–39.7%), 30.6% (95% CI: 28.1–33.1%); PFS was 45.4% (95% CI: 43.8–47.0%), 31.4% (95% CI: 29.5–33.4%), 24.7% (95% CI: 22.3–27.2%), respectively. The observed improvements in OS and PFS rates can partially be explained by a significant decrease of cumulative relapse incidences (Table 4, Fig. 3). Main causes of death after allo-HSCT were disease-related/relapse in 44.7% of cases followed by transplant--related causes, mostly infectious complications (35.8%), GvHD (12.9%), other/unknown causes (7.1%) and secondary malignancies (0.3%). We observed a significant decrease in NRM after 1999 (Table 4, Fig. 3). For acute and chronic GvHD incidences, a significant decrease over time was noted (Table 4, Fig. S3). No significant differences between matched-related (sibling and non-sibling) and unrelated donor transplants in terms of severe acute GvHD Grades III-IV at day 100 [8.2% (95% CI: 6.9–9.7%) vs. 8.5% (95% CI: 7.2–10.0%), *p* = 0.926] were observed. The incidences of chronic GvHD at 1 year were also comparable for patients with matched-related and unrelated donors with 24.1% (95% CI: 22.0–26.3%) and 23.2% (95% CI: 21.1–25.3%) (*p* = 0.833), respectively. Haploidentical/mismatched-related transplantations became more common since 2010 ( > 5% of allo-HSCT) with incidences of acute GvHD grades III–IV at day 100 being 12.6% (95% CI: 9.4–16.2%) and 16.7% (95% CI: 13.1–20.7%) for chronic GvHD at 1 year, comparing favorably to patients who received matched-related or unrelated donor transplants [acute GvHD grades III–IV at day 100: 8.4% (95% CI: 7.4–9.4%), *p* = 0.027; chronic GVHD at 1 year: 23.7% (95% CI: 22.2–35.2%), *p* = 0.002]. To evaluate the effect of disease status and conditioning intensity we used a multivariate model that incorporated year of HSCT, age at HSCT, gender, time interval from diagnosis to HSCT ≤ 12 months, disease status at HSCT, and conditioning intensity (Table S3). This model revealed that disease status at allo-HSCT, most notably CR, as well as a longer interval between diagnosis and HSCT ( > 12 months) were associated with superior OS and PFS rates. These observations reflect a higher risk of relapse for patients not in CR at allo-HSCT (*p* < 0.001) and a reduced risk of relapse in patients allografted >12 months from diagnosis [HR = 0.75 (95% CI: 0.65–0.85), *p* < 0.001]. Conditioning with MAC protocols was associated with inferior PFS compared to RIC conditioning [HR = 1.23 (95% CI: 1.12–1.34), *p* < 0.001] and OS [HR = 1.34 (95% CI: 1.22–1.47), *p* < 0.001] in the model. The risk of NRM significantly increased with age at allo-HSCT [HR = 1.12 (95% CI: 1.08–1.15), *p* < 0.001], and conditioning intensity [HR = 1.32 (95% CI: 1.14–1.53), *p* < 0.001] (Table S3).

**Table 4:** Outcomes after allo-HSCT by time periods.

Outcomes after allo-HSCT in % (95% CI)								
Variable	Time	Overall	Time Periods					
			1990–1999	2000–2004	2005–2009	2010–2015	2015–2021	*P* value
NRM	1 year	20.1 (18.8–21.3)	32.7 (26.3–39.3)	21.5 (17.9–25.3)	21 (17.9–24.4)	18.8 (16.4–21.2)	18.6 (16.7–20.5)	<0.001
	3 years	23.8 (22.5–25.2)	34.9 (28.3–41.6)	24.1 (20.4–28.1)	23.8 (20.4–27.3)	23.5 (20.9–26.2)	22.6 (20.5–24.8)	
RI	1 year	34.5 (33–36.1)	34.4 (27.8–41)	34.5 (30.2–38.8)	39.5 (35.6–43.4)	34.2 (31.3–37.1)	32.9 (30.5–35.2)	0.008
	3 years	40 (38.4–41.5)	37.6 (30.8–44.3)	41.2 (36.7–45.6)	44.7 (40.6–48.7)	38.6 (35.6–41.6)	38.9 (36.4–41.4)	
PFS	1 year	45.4 (43.8–47.0)	32.9 (26.4–39.5)	44 (39.5–48.5)	39.4 (35.5–43.4)	47 (43.9–50)	48.6 (46–51)	<0.001^b^
	2 years	38.4 (36.8–40.0)	28.6 (22.5–35.1)	37.5 (33.2–41.9)	33 (29.2–36.9)	40.3 (37.2–43.3)	40.8 (38.2–43.3)	
	3 years	36.2 (34.6–37.8)	27.5 (21.5–33.9)	34.7 (30.4–39)	31.5 (27.7–35.3)	37.9 (34.8–40.9)	38.5 (36.0–41.0)	
	5 years	31.4 (29.5–33.4)	23.6 (17.8–29.8)	31 (26.9–35.3)	27.7 (24–31.4)	35.5 (32.5–38.6)	^a^	
	10 years	24.7 (22.3–27.2)	21.6 (16.1–27.8)	27.8 (23.7–32)	23.2 (19.7–26.9)	^a^	^a^	
OS	1 year	54.5 (53.0–56.0)	39.4 (33.2–45.6)	52.4 (48.1–56.5)	49.1 (45.2–52.8)	56.2 (53.2–59.1)	58.2 (55.8–60.5)	<0.001^b^
	2 years	46.5 (44.9–48)	35.9 (29.8–42)	46.3 (42–50.4)	42.1 (38.3–45.8)	46.7 (43.7–49.7)	49.4 (46.9–51.8)	
	3 years	43.2 (41.6–44.7)	33.2 (27.2–39.2)	42.6 (38.4–46.8)	38.5 (34.8–42.3)	43.8 (40.8–46.8)	46.1 (43.6–48.6)	
	5 years	37.8 (35.8–39.7)	29.8 (24–35.8)	39.2 (35–43.3)	34.2 (30.6–38)	40.9 (37.9–43.9)	^a^	
	10 years	30.6 (28.1–33.1)	28.2 (22.5–34.2)	33.1 (28.9–37.2)	29.3 (25.6–33)	^a^	^a^	
aGvHD Grade III–IV	100 days	9 (8.1–10)	16.2 (11–22.2)	7.5 (5.2–10.3)	5.6 (3.8–7.8)	6.1 (4.7–7.7)	11.7 (10.1–13.4)	<0.001
cGVHD	1 year	22.9 (21.5–24.3)	17.9 (12.6–23.9)	21.2 (17.4–25.3)	22.2 (18.8–25.8)	25.9 (23.1–28.7)	22.1 (20–24.3)	0.006
	2 years	27.2 (25.8–28.7)	20.1 (14.6–26.3)	26.3 (22.1–30.7)	27.2 (23.5–31.1)	29.9 (27–32.9)	26.4 (24.2–28.7)	

*CI* confidence interval, *NRM* non-relapse mortality, *RI* relapse incidence, *PFS* Progression-free Survival, *OS* Overall Survival, *aGvHD* acute graft-versus-host disease, *cGvHD* chronic graft-versus-host disease.

^a^Outcomes were not available due to follow-up.

^b^*P* value representing outcomes with available data.

## Discussion

In the current study, we describe the evolution of transplantation activities and modalities, and provide outcome data for more than 40,000 transplants performed for DLBCL covering 32 years. We observed steadily increasing numbers of autologous and allogeneic HSCT with a growing proportion of transplants being performed in CR or PR. After 2018, a steep decline in transplantation activities was observed coinciding with the availability of CAR T-cells and other new agents entering the clinical arena. Although we cannot exclude that the SARS-CoV-2 pandemic temporarily disrupted transplant activities also for DLBCL, there is evidence based on the most recent numbers reported to EBMT that post-pandemic transplant numbers continue to decrease [32, 33]. The promising reports on CAR T-cell therapies including the recently published randomized studies comparing CAR T-cells to auto-HSCT for treatment of DLBCL patients failing first-line therapy will further boost CAR T-cell therapy [24, 26]. The approval of bispecific antibodies and antibody-drug-conjugates will accelerate this trend [34–36].

Clinical characteristics of transplant patients evolved from younger patients with advanced disease towards an older but medically fit population undergoing transplantation in CR or PR. Changes in transplant modalities like the use of mobilized blood as the source of hematopoietic stem cells for auto- and allo-HSCT and the adoption of RIC prior to allo-HSCT fostered easier access to transplantation also for older and frail patients. The switch from HLA-identical sibling donors to unrelated, and, more recently, haploidentical donors paralleled by changes in GvHD prophylaxis also contributed to the increase in allogeneic transplantation numbers.

Until today, there is no consensus on the optimal preparatory regimen for auto- or allo-HSCT [12, 13, 16, 17, 37–39]. BEAM remains the most commonly used conditioning regimen prior to auto-HSCT, while busulfan/fludarabine and TBI-based protocols were most frequently used to prepare for allo-HSCT. TBI-based protocols using doses >6 Gy clearly decreased from 75% in the 1990s to 9% in the most recent period, underscoring the transition to mainly chemotherapy-based RIC. The most popular MAC protocols combined busulfan, fludarabine and TBI, while the most popular RIC regimens were fludarabine/melphalan and busulfan/fludarabine at lower doses. Because patients prepared with MAC tend to present with higher tumor burden and more advanced disease, direct comparisons of outcomes after RIC and MAC remain problematic. The reported 3-year NRM rate of approximately 24% aligns with previous findings that myeloablative conditioning (MAC) regimens are associated with higher NRM [16, 40, 41]. Despite the increasing use of RIC over time, in this analysis, over 40% of allo-HSCTs performed since 2010 were preceded by MAC conditioning. This likely has contributed to the relatively high NRM rates observed [42, 43]. The increasing usage of RIC regimens over time was also paralleled by more patients undergoing allo-HSCT in CR or PR and reflects the investigators’ views that RIC is the preferred conditioning approach for patients with less rapidly growing tumors and lower tumor burden at transplantation. For older and frail patients, RIC frequently is the only option to prepare patients for allo-HSCT. OS and PFS as the most important outcome parameters significantly improved over time. This seems at least partly due to a decrease in relapse rates, which in turn mirrored the lower number of patients transplanted with active disease. The 3-year OS- and PFS rates of approximately 45% and 38% for the most recent period appear slightly better than those reported for the only randomized trial involving allo-HSCT for DLBCL (42% and 39%, respectively) [16] but are in line with other registry-based analyses, which consistently showed 3-year PFS rates of 30–40% [13, 41, 44].

NRM rates after allo-HSCT and auto-HSCT showed a trend to decline after 1999. Improvements in recipient/donor matching, conditioning, GvHD prophylaxis and supportive care obviously were able to compensate for the increasing patient age and broader donor selection. Patients who received an auto-HSCT for consolidation of a first CR or PR showed the highest survival rates approaching 80%. Despite such excellent outcomes, consolidative auto-HSCT cannot generally be recommended after several randomized studies failed to demonstrate an improvement of PFS or OS in young, high-risk patients with DLBCL when compared to conventional immunochemotherapy [45–47].

In the early days, a large proportion of allo-HSCT were performed in patients with active disease conditioned with myeloablative regimens [48, 49]. These transplants were characterized by high relapse and NRM rates resulting in poor survival. In recent years, allo-HSCT frequently was performed in responding patients after reduced-intensity conditioning (RIC) featuring lower NRM and RI resulting in better survival [12, 13]. A large proportion of patients underwent allo-HSCT after failing auto-HSCT with results comparable to those reported for patients without a previous autograft [13, 49].

The observed parallel increase in GvHD prophylaxis using post-transplantation cyclophosphamide (PTCy) and haploidentical donors after 2015 is likely attributable to encouraging results highlighting comparable survival rates for haploidentical donors as compared to matched sibling or unrelated donors [50–52]. Recently, a large phase 3 trial demonstrated superior outcomes with PTCy-based GvHD prophylaxis in patients, predominantly with leukemia/MDS ( > 85%), undergoing allo-HCT after reduced-intensity conditioning (RIC) with either an HLA-matched or 7/8-mismatch donor. Although these findings may apply also to DLBCL patients, no formal proof is available and studies addressing this point are unlikely to be conducted, given the recent decline of allo-HCT in patients with DLBCL [53].

The present study has limitations. Most importantly, we cannot know how many patients initially deemed transplant candidates could not make it to transplantation because of disease progression or toxicity of salvage therapies. Unknown confounders of outcomes after transplantation might have gone undetected, and we cannot directly compare outcomes after HSCT with outcomes of patients receiving alternative treatments. Additionally, due to changes in data reporting over time not all details of pre-transplant treatments may have been caught, particularly for patients transplanted in the early years of data collection. While we acknowledge these and other limitations of any retrospective analysis, we believe that the huge number of patients closely followed for up to 32 years provides a solid foundation for further discussion of evolving treatment strategies in DLBCL.

Our analysis reflects the changing role of transplantation in the treatment of DLBCL. Allo-HSCT, CAR-T cells, and bispecific antibodies represent different forms of immunotherapy. Therefore, we suggest that international databases like ours should be open to include all these modalities, hopefully also allowing for comparison of competing strategies. The large body of data presented here summarizes long-term outcomes after auto- and allo-HSCT, which may serve as real-world benchmarks when comparing transplantation to newer, more targeted therapies.

### Supplementary information

Supplemental Material

## Data Availability

The datasets presented in the study are included in the article/Supplementary information. Further inquiries can be directed to the corresponding author.
