# Supplementary material for: Hematopoietic stem cell transplantation for DLBCL: a report from the European Society for Blood and Marrow Transplantation on more than 40,000 patients over 32 years
Source: Blood Cancer J. 2024 Jul 5;14(1):106. doi: 10.1038/s41408-024-01085-9 (PMC11226679; doi:10.1038/s41408-024-01085-9)
Supplement: Supplementary file 1 — Supplemental Material [file 41408_2024_1085_MOESM1_ESM.docx]

**Supplementary** **Information**

**Hematopoietic Stem Cell Transplantation for DLBCL:**

**A Report from the European Society for Blood and Marrow Transplantation on more than 40,000 Patients over 32 Years**

Philipp Berning^1^, Mathilde Fekom^2^, Maud Ngoya^2^, Anthony H. Goldstone^3^, Peter Dreger^4^, Silvia Montoto^5^, Hervé Finel^2^, Evgenii Shumilov^1^, Patrice Chevallier^6^, Didier Blaise^7^, Tim Strüssmann^8^, Ben Carpenter^9^, Edouard Forcade^10^, Cristina Castilla-Llorente^11^, Marek Trneny^12^, Hervé Ghesquieres^13^, Saveria Capria^14^, Catherine Thieblemont^15^, Igor Wolfgang Blau^16^, Ellen Meijer^17^, Annoek E.C. Broers^18^, Anne Huynh^19^, Denis Caillot^20^, Wolf Rösler^21^, Stephanie Nguyen Quoc^22^, Jörg Bittenbring^23^, Arnon Nagler^24^, Jacques-Emmanuel Galimard^2^, Bertram Glass^25^, Anna Sureda^26#^, Norbert Schmitz^1#^

1. Department of Hematology and Oncology, University Hospital Muenster, Muenster, Germany
2. European Society for Blood and Marrow Transplantation, Paris, France
3. HCA Healthcare, Macmillan Cancer Centre, London, United Kingdom
4. Department of Medicine V, University of Heidelberg, Heidelberg, Germany
5. St. Bartholomew's Hospital, Barts Health NHS Trust, London, United Kingdom
6. Department of Hematology, CHU Nantes, Nantes, France
7. Transplantation and Cellular Immunotherapy Program, Department of Hematology, Instititut Paoli Calmettes, MSC Lab, Aix Marseille University, Marseille, France
8. Department of Hematology, Oncology and Stem Cell Transplantation, University of Freiburg Medical Center, Freiburg, Germany
9. Department of Hematology, University College London Hospitals, London, United Kingdom
10. Service d'Hématologie Clinique et Thérapie Cellulaire, CHU Bordeaux, F-33000, Bordeaux, France
11. Department of Hematology, Gustave Roussy Cancer Campus, Villejuif, France
12. First Faculty of Medicine, Charles University, Praha, Czech Republic
13. Hospices Civils de Lyon, Hôpital Lyon Sud, Service d'Hématologie, Pierre Bénite, France.
14. Department of Translational and Precision Medicine, Policlinico Umberto I Hospital, Sapienza University of Rome, Rome, Italy
15. Department of Hemato-Oncology, Hôpital Saint-Louis APHP, Paris, France
16. Charité-Universitätsmedizin Berlin, Freie Universität Berlin and Humboldt-Universität Berlin, Department of Hematology, Oncology and Tumor Immunology, Campus Virchow Clinic, Berlin, Germany
17. Department of Hematology, Amsterdam University Medical Center, Free University, Amsterdam, the Netherlands
18. Department of Hematology, Erasmus MC Cancer Institute, Rotterdam, Netherlands.
19. CHU - Institut Universitaire du Cancer Toulouse, Oncopole, I.U.C.T-O, Toulouse, France
20. Department of Hematology, CHU Dijon, Dijon, France
21. Department of Internal Medicine 5, Hematology and Oncology, University of Erlangen-Nuremberg, Erlangen, Germany
22. Department of Hematology, AP-HP, Sorbonne Université, Pitié- Salpêtrière Hospital, Paris, France
23. Department of Hematology and Oncology, Saarland University Medical School, Homburg, Germany
24. Division of Hematology, Sheba Medical Center, Tel Hashomer, Israel
25. Department of Hematology, Oncology, Tumor Immunology, and Palliative Care, Helios Klinikum Berlin-Buch, Berlin, Germany
26. Department of Hematology, Institut Català d'Oncologia Hospitalet, IDIBELL, Universitat de Barcelona, Barcelona, Spain

^#^ A.S. and N.S. contributed equally to this work.

Presented in abstract form at the 65^th^ annual meeting of the American Society of Hematology in San Diego, CA, December 9-12, 2023. P.B. received a 2023 ASH Abstract Achievement Award.

**Corresponding author:** Norbert Schmitz, Department of Hematology and Oncology, University Hospital Muenster, Albert-Schweitzer-Campus 1 A1, 48149 Muenster, Germany; Phone: +49 251 8347587; Email: [norbert.schmitz@ukmuenster.de](mailto:norbert.schmitz@ukmuenster.de)

**Supplementary Figures**

**Figure S1.** **CONSORT flowchart.**

CONSORT = Consolidated Standards of Reporting Trials; HSCT = hematopoietic stem cell transplantation; allo-HSCT = allogeneic HSCT; auto-HSCT = autologous HSCT.

**Figure S2.** **Kaplan-Meier estimates of outcomes for auto-HSCT by treatment lines and disease status.**

Overall survival and progression-free survival for patients who underwent auto-HSCT (**A**) after ≤1 line, 2 lines or ≥3 lines of treatment in CR (**B**) in CR or PR after any lines of treatment. Auto -HSCT = autologous hematopoietic stem cell transplantation; CR = complete remission; PR = partial remission.

**Figure S3. Cumulative incidences of acute and chronic GvHD over time.**

(**A**) Acute GvHD grades 3–4 and (B) chronic GvHD over by 5-year periods between 1990 and 2019 (GvHD outcomes for the 1990-1999 period are summarized due to low event numbers between 1990-1994). GvHD = graft-versus-host disease.

**Figure S1. CONSORT flowchart.**

**
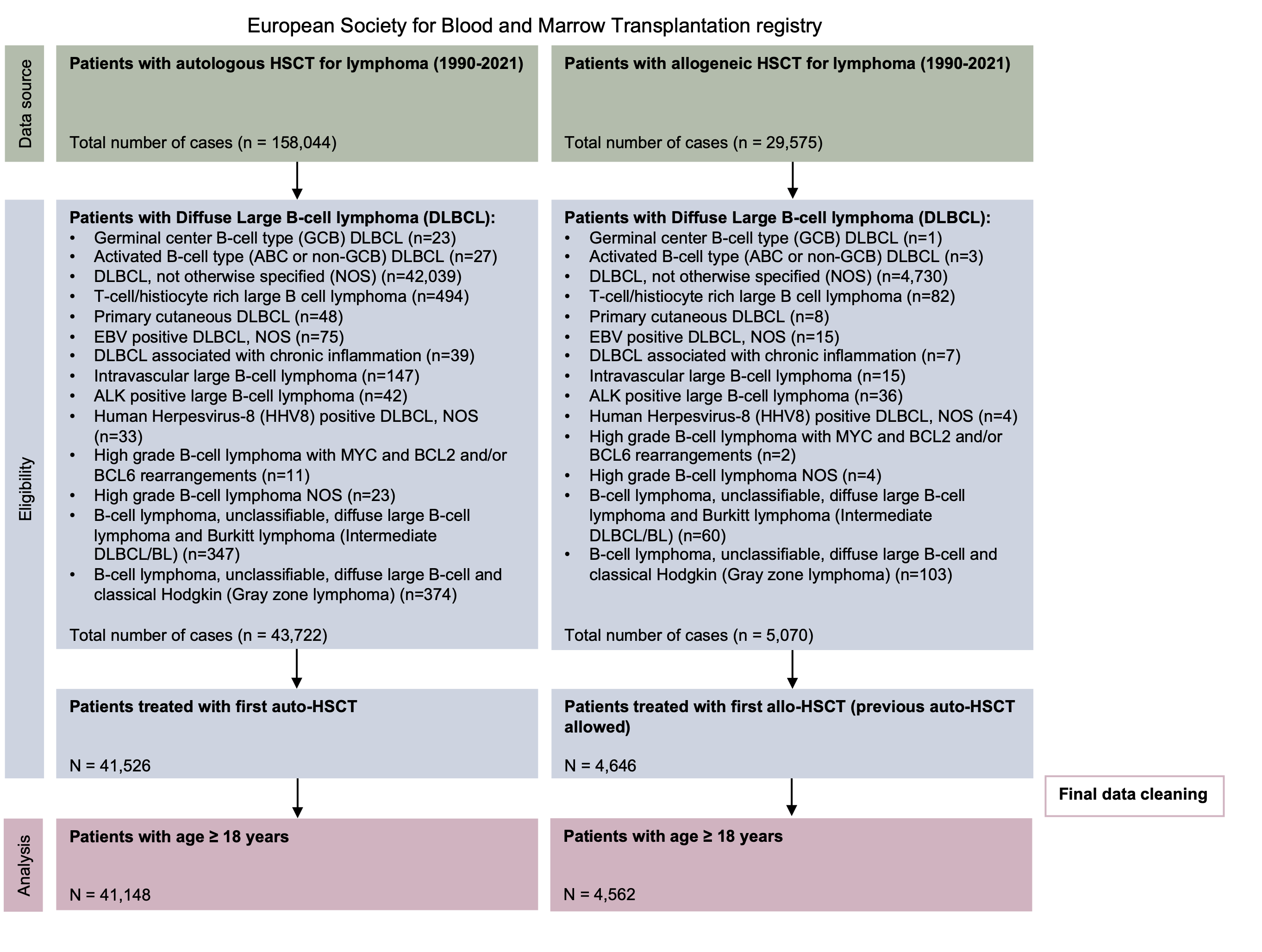
**

**Figure S2. Kaplan-Meier estimates of outcomes for auto-HSCT by treatment lines and disease status.**

**
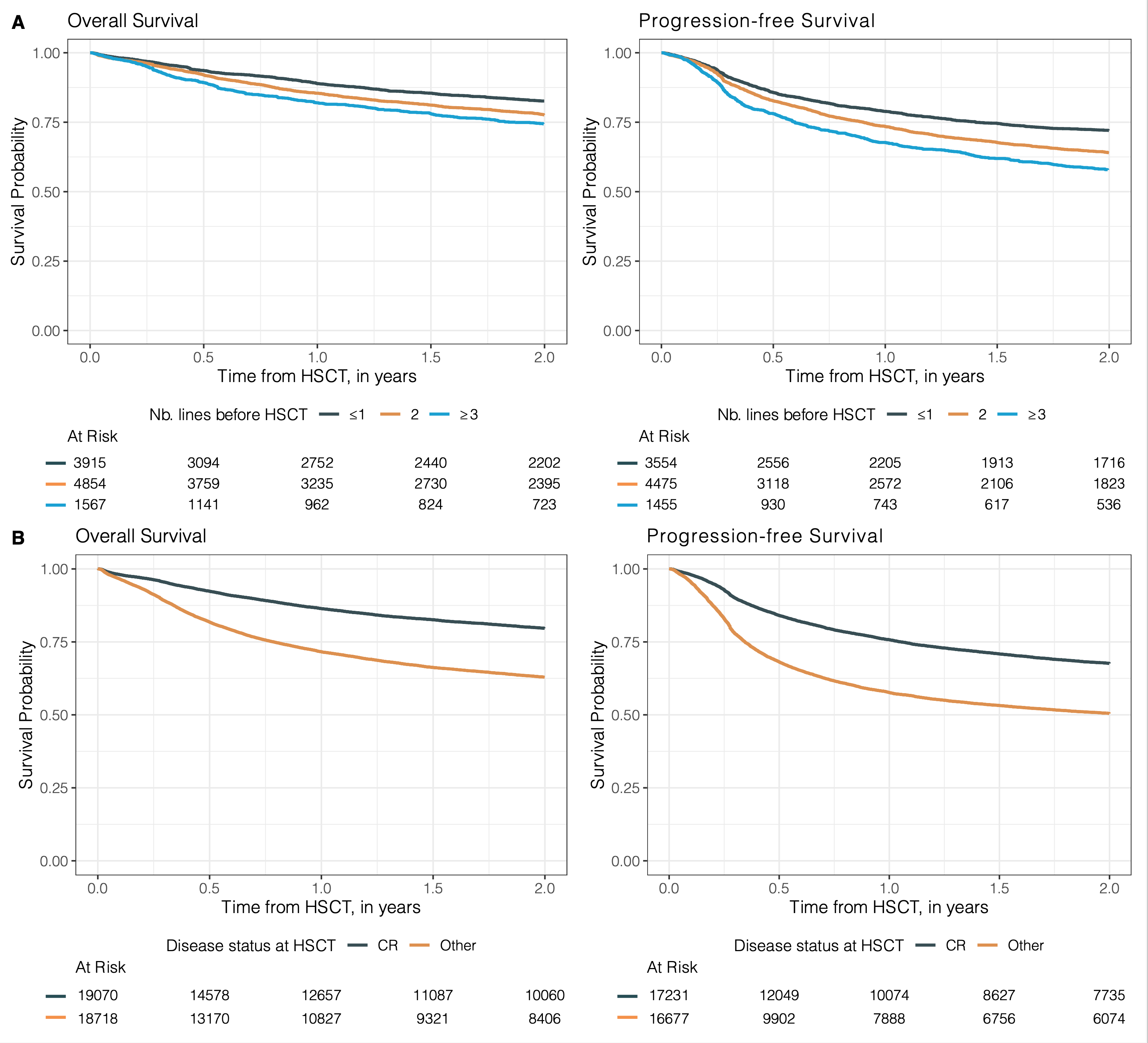
**

**Figure S3. Cumulative incidences of acute and chronic GvHD over time.**

**
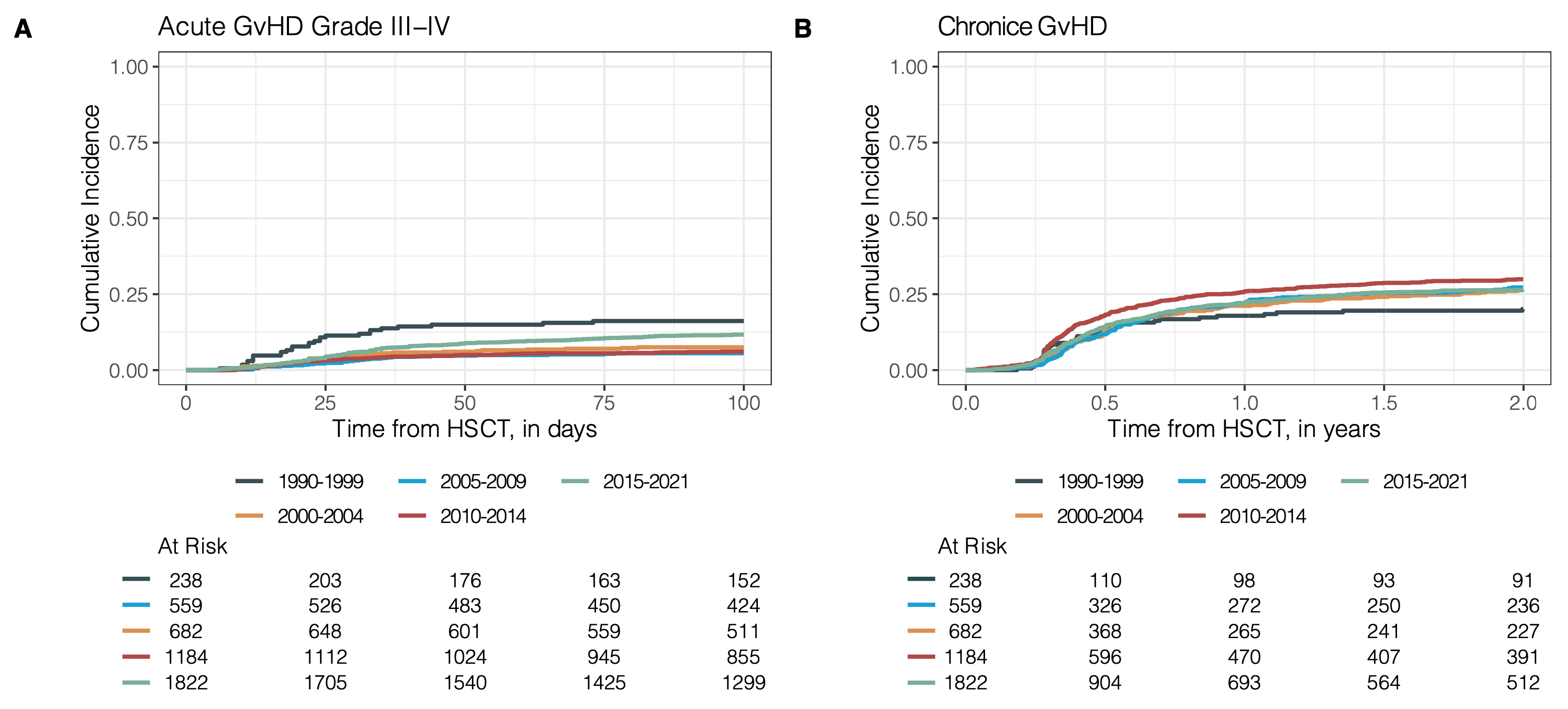
**

**Table S1. Contributing centers and patient numbers.**

| **Centers** | ***N*** |
| --- | --- |
| Nantes [Hotel Dieu] | 578 |
| Marseille [Paoli Calmettes] | 571 |
| Freiburg [University] | 470 |
| London [UCL] | 407 |
| Paris [Pitie-Salpetriere] | 371 |
| Pessac [H Haut-Leveque] | 351 |
| Villejuif [Gustave Roussy] | 348 |
| Paris [St Louis, Adulte] | 346 |
| Brescia [Spedali Civili] | 343 |
| Amsterdam [VU Univ Med Ctr] | 325 |
| Rotterdam [Erasmus MC] | 322 |
| Toulouse [H Purpan] | 321 |
| Rome [Emat, La Sapienza] | 303 |
| Lyon [Lyon-Sud] | 287 |
| Berlin [Charité Univ] | 286 |
| Heidelberg [Medizinische Kl] | 285 |
| Prague [Charles U H, Med] | 280 |
| Milano [Trap Mid Osseo] | 269 |
| Bologna [S Orsola-Malpighi] | 267 |
| Brussels [St. Luc] | 264 |
| Strasbourg [H Hautepierre] | 263 |
| Rouen [Becquerel] | 261 |
| Katowice [Silesian Med Acad] | 256 |
| Muenster [University] | 256 |
| Rome [Tor Vergata] | 250 |
| Paris [St Antoine] | 250 |
| Vandoeuvre Les Nancy [Hosp] | 246 |
| Bergamo [Ospedale, ematol] | 245 |
| Kiel [UKSH] | 243 |
| Bern [221] | 240 |
| Leeds [St James] | 240 |
| Haifa [Rambam MCH] | 239 |
| Mainz [Johannes-Gutenberg] | 237 |
| Caen [Hopital, Hematol] | 236 |
| Groningen [Univ H] | 228 |
| Tel-Hashomer [Univ Adults] | 228 |
| London [Kings College H] | 228 |
| Udine [Univ H] | 227 |
| Tours [H Bretonneau] | 226 |
| Birmingham [Queen Elizabeth] | 225 |
| Cologne [Univ, Medicine] | 225 |
| Montpellier [University] | 225 |
| Southampton [General H] | 222 |
| Nottingham [City H] | 220 |
| Manchester [Christie] | 218 |
| Gliwice [Sklodowska] | 217 |
| Stockholm [Univ H] | 216 |
| Dresden [Universitaets Kl] | 216 |
| Budapest [National Med Ctr] | 214 |
| Brest [C.H.R.U Brest] | 214 |
| Salamanca [H Clinico] | 211 |
| Lausanne [820] | 211 |
| Lyon [Leon Berard] | 209 |
| Glasgow [Royal Infirmary] | 209 |
| Lund [Univ H] | 209 |
| Newcastle-Upon-Tyne [Freeman H] | 207 |
| Maastricht [Univ H] | 206 |
| Torino [S. Giovanni (CTO)] | 205 |
| Lille [H Claude Huriez] | 203 |
| Rennes [H Sud/Pontchaillou] | 202 |
| Goeteborg [Sahlgrenska Univ H] | 201 |

**Table S2. Multivariate Cox proportional hazards model for auto-HSCT patients.**

|  | **Relapse Incidence** | | | **Non-Relapse Mortality** | | | **Progression-free Survival** | | | **Overall Survival** | | |
| --- | --- | --- | --- | --- | --- | --- | --- | --- | --- | --- | --- | --- |
| **Variable** | **HR** | **95%-CI** | ***P* value** | **HR** | **9****5%-CI** | ***P* value** | **HR** | **95%-CI** | ***P* value** | **HR** | **95%-CI** | ***P* value** |
| **Year group HSCT** | | | | | | | | | | | | |
| 2015-2021 | Ref. |  |  | Ref. |  |  | Ref. |  |  |  | Ref. |  |
| 2010-2014 | 0.95 | 0.89, 1.00 | 0.051 | 1.03 | 0.90, 1.19 | 0.7 | 0.96 | 0.91, 1.01 | 0.091 | 1.07 | 1.01, 1.14 | **0.031** |
| 2005-2009 | 0.98 | 0.92, 1.04 | 0.5 | 1.06 | 0.89, 1.25 | 0.5 | 0.99 | 0.93, 1.05 | 0.7 | 1.1 | 1.02, 1.18 | **0.012** |
| 2000-2004 | 1.02 | 0.94, 1.11 | 0.6 | 1.22 | 0.99, 1.50 | 0.058 | 1.04 | 0.97, 1.12 | 0.3 | 1.15 | 1.05, 1.25 | **0.002** |
| 1995-1999 | 1.25 | 1.14, 1.37 | **<0.001** | 1.35 | 1.05, 1.72 | **0.018** | 1.26 | 1.16, 1.37 | **<0.001** | 1.29 | 1.17, 1.42 | **<0.001** |
| 1990-1994 | 1.53 | 1.36, 1.73 | **<0.001** | 2.12 | 1.56, 2.88 | **<0.001** | 1.59 | 1.42, 1.78 | **<0.001** | 1.7 | 1.50, 1.94 | **<0.001** |
| **Age at HSCT (continuous variable)** | | | | | | | | | | | | |
| By 5 years | 1.05 | 1.04, 1.06 | **<0.001** | 1.18 | 1.15, 1.22 | **<0.001** | 1.06 | 1.05, 1.07 | **<0.001** | 1.09 | 1.08, 1.10 | **<0.001** |
| **Patient gender** | | | | | | | | | | | | |
| Male | Ref. |  |  | Ref. |  |  | Ref. |  |  |  | Ref. |  |
| Female | 0.94 | 0.90, 0.98 | **0.006** | 1.01 | 0.91, 1.13 | 0.8 | 0.95 | 0.91, 0.99 | **0.014** | 0.93 | 0.89, 0.98 | **0.005** |
| **Time from Diagnosis to HSCT** | | | | | | | | | | | | |
| ≤12 months | Ref. |  |  | Ref. |  |  | Ref. |  |  |  | Ref. |  |
| > 12 months | 1.2 | 1.15, 1.26 | **<0.001** | 1.28 | 1.14, 1.44 | **<0.001** | 1.22 | 1.16, 1.27 | **<0.001** | 1.08 | 1.03, 1.13 | **0.002** |
| **Disease status at auto-HSCT** | | | | | | | | | | | | |
| CR | Ref. |  |  | Ref. |  |  | Ref. |  |  |  | Ref. |  |
| PR | 1.61 | 1.53, 1.69 | **<0.001** | 1.1 | 0.97, 1.25 | 0.12 | 1.53 | 1.46, 1.60 | **<0.001** | 1.58 | 1.50, 1.67 | **<0.001** |
| SD / PD | 2.98 | 2.81, 3.16 | **<0.001** | 1.73 | 1.49, 2.01 | **<0.001** | 2.76 | 2.62, 2.92 | **<0.001** | 3.26 | 3.07, 3.47 | **<0.001** |
| **Conditioning Regimen** | | | | | | | | | | | | |
| BEAM or similar | Ref. |  |  | Ref. |  |  | Ref. |  |  |  | Ref. |  |
| Other | 1.11 | 1.06, 1.17 | **<0.001** | 1.37 | 1.22, 1.55 | **<0.001** | 1.14 | 1.09, 1.20 | **<0.001** | 1.24 | 1.18, 1.31 | **<0.001** |
| Abbreviations: HR= hazard ratio; CI= confidence interval; Ref.= Reference group. HSCT=hematopoietic stem cell transplantation; CR= complete remission; PR= partial response; SD= stable disease; PD= progressive disease. | | | | | | | | | | | | |

**Table S3. Multivariate Cox proportional hazards model for allo-HSCT patients.**

|  | **Relapse Incidence** | | | **Non-Relapse Mortality** | | | **Progression-free Survival** | | | **Overall Survival** | | |
| --- | --- | --- | --- | --- | --- | --- | --- | --- | --- | --- | --- | --- |
| **Variable** | **HR** | **95%-CI** | ***P* value** | **HR** | **95%-CI** | ***P* value** | **HR** | **95%-CI** | ***P* value** | **HR** | **95%-CI** | ***P* value** |
| **Year group HSCT** | | | | | | | | | | | | |
| 2015-2021 | Ref. |  |  | Ref. |  |  | Ref. |  |  |  | Ref. |  |
| 2010-2014 | 0.95 | 0.83, 1.08 | 0.4 | 1.09 | 0.92, 1.30 | 0.3 | 1 | 0.90, 1.11 | >0.9 | 1.08 | 0.97, 1.21 | 0.2 |
| 2005-2009 | 1.13 | 0.96, 1.31 | 0.13 | 1.2 | 0.97, 1.48 | 0.1 | 1.15 | 1.01, 1.30 | **0.03** | 1.19 | 1.05, 1.36 | **0.008** |
| 2000-2004 | 0.88 | 0.73, 1.05 | 0.2 | 1.2 | 0.94, 1.54 | 0.14 | 0.98 | 0.85, 1.14 | 0.8 | 1.07 | 0.92, 1.24 | 0.4 |
| 1990-1999* | 0.72 | 0.54, 0.98 | **0.036** | 1.95 | 1.40, 2.72 | **<0.001** | 1.06 | 0.85, 1.32 | 0.6 | 1.24 | 1.00, 1.55 | 0.053 |
| **Age at HSCT (continuous variable)** | | | | | | | | | | | | |
| By 5 years | 1 | 0.98, 1.02 | >0.9 | 1.12 | 1.08, 1.15 | **<0.001** | 1.04 | 1.02, 1.06 | **<0.001** | 1.06 | 1.04, 1.08 | **<0.001** |
| **Patient gender** | | | | | | | | | | | | |
| Male | Ref. |  |  | Ref. |  |  | Ref. |  |  |  | Ref. |  |
| Female | 0.96 | 0.85, 1.07 | 0.4 | 0.99 | 0.86, 1.15 | >0.9 | 0.97 | 0.89, 1.06 | 0.5 | 0.96 | 0.87, 1.05 | 0.4 |
| **Time from Diagnosis to HSCT** | | | | | | | | | | | | |
| ≤12 months | Ref. |  |  | Ref. |  |  | Ref. |  |  |  | Ref. |  |
| > 12 months | 0.75 | 0.67, 0.85 | **<0.001** | 1.15 | 0.97, 1.37 | 0.11 | 0.87 | 0.79, 0.96 | **0.005** | 0.92 | 0.83, 1.02 | 0.1 |
| **Disease status at HSCT** | | | | | | | | | | | | |
| CR | Ref. |  |  | Ref. |  |  | Ref. |  |  |  | Ref. |  |
| PR | 1.86 | 1.61, 2.14 | **<0.001** | 1.25 | 1.05, 1.49 | **0.014** | 1.58 | 1.42, 1.77 | **<0.001** | 1.59 | 1.42, 1.79 | **<0.001** |
| SD / PD | 3.18 | 2.79, 3.63 | **<0.001** | 1.49 | 1.25, 1.77 | **<0.001** | 2.4 | 2.16, 2.67 | **<0.001** | 2.34 | 2.10, 2.61 | **<0.001** |
| **Conditioning Intensity** | | | | | | | | | | | | |
| RIC | Ref. |  |  | Ref. |  |  | Ref. |  |  |  | Ref. |  |
| MAC | 1.18 | 1.05, 1.32 | **0.004** | 1.32 | 1.14, 1.53 | **<0.001** | 1.23 | 1.12, 1.34 | **<0.001** | 1.34 | 1.22, 1.47 | **<0.001** |
| Abbreviations: HR= hazard ratio; CI= confidence interval; Ref.= Reference group. HSCT=hematopoietic stem cell transplantation; CR= complete remission; PR= partial response; SD= stable disease; PD= progressive disease.  * Outcomes were summarized between 1990 and 1999 because of lower number of events. | | | | | | | | | | | | |
